# Effects of Dietary Protein and Fiber at Breakfast on Appetite, *ad Libitum* Energy Intake at Lunch, and Neural Responses to Visual Food Stimuli in Overweight Adults

**DOI:** 10.3390/nu8010021

**Published:** 2016-01-05

**Authors:** R. Drew Sayer, Akua F. Amankwaah, Gregory G. Tamer, Ningning Chen, Amy J. Wright, Jason R. Tregellas, Marc-Andre Cornier, David A. Kareken, Thomas M. Talavage, Megan A. McCrory, Wayne W. Campbell

**Affiliations:** 1 Department of Nutrition Science, Purdue University, West Lafayette, IN 47907, USA; sayer@purdue.edu (R.D.S.); aboaten@purdue.edu (A.F.A.); amyjwright2@purdue.edu (A.J.W.); 2 Weldon School of Biomedical Engineering, Purdue University, West Lafayette, IN 47907, USA; gtamer@purdue.edu (G.G.T.); tmt@purdue.edu (T.M.T.); 3 Department of Statistics, Purdue University, West Lafayette, IN 47907, USA; chen929@purdue.edu; 4 Department of Psychiatry, University of Colorado School of Medicine, Anschutz Medical Campus, Aurora, CO 80045, USA; Jason.Tregellas@ucdenver.edu; 5 Department of Medicine, Division of Endocrinology, Metabolism and Diabetes, University of Colorado School of Medicine, Anschutz Medical Campus, Aurora, CO 80045, USA; Marc.Cornier@ucdenver.edu; 6 Department of Neurology, Indiana University School of Medicine, Indianapolis, IN 46202, USA; dkareken@iu.edu; 7 Department of Nutrition, Georgia State University, Atlanta, GA 30303, USA; mmccrory@gsu.edu

**Keywords:** fMRI, food reward, overweight, appetite regulation, dietary protein, dietary fiber

## Abstract

Increasing either protein or fiber at mealtimes has relatively modest effects on ingestive behavior. Whether protein and fiber have additive or interactive effects on ingestive behavior is not known. Fifteen overweight adults (5 female, 10 male; BMI: 27.1 ± 0.2 kg/m^2^; aged 26 ± 1 year) consumed four breakfast meals in a randomized crossover manner (normal protein (12 g) + normal fiber (2 g), normal protein (12 g) + high fiber (8 g), high protein (25 g) + normal fiber (2 g), high protein (25 g) + high fiber (8 g)). The amount of protein and fiber consumed at breakfast did not influence postprandial appetite or *ad libitum* energy intake at lunch. In the fasting-state, visual food stimuli elicited significant responses in the bilateral insula and amygdala and left orbitofrontal cortex. Contrary to our hypotheses, postprandial right insula responses were lower after consuming normal protein *vs.* high protein breakfasts. Postprandial responses in other *a priori* brain regions were not significantly influenced by protein or fiber intake at breakfast. In conclusion, these data do not support increasing dietary protein and fiber at breakfast as effective strategies for modulating neural reward processing and acute ingestive behavior in overweight adults.

## 1. Introduction

Obesity continues to be a major public health concern in the United States with nearly 70% of adults being overweight or obese (BMI ≥ 25.0 kg/m^2^) according to the National Health and Nutrition Examination Survey (NHANES) 2011–2012 [1]. Several prospective studies demonstrate that those with an overweight BMI are at risk of continued weight gain and progression to obesity [2,3,4]. Therefore, lifestyle interventions designed to prevent further weight gain in overweight individuals could have a significant public health impact by preventing obesity-related chronic morbidities [5] and mortality [6].

Increasing the amount of dietary protein at breakfast is one such potential intervention. Higher protein diets are associated with modestly decreased adiposity [7], and meta-analytical results from randomized clinical trials demonstrate greater weight loss with high protein *vs.* standard protein diets [8]. The observed effects of higher protein diets on body weight and/or adiposity are likely partially explained by enhanced postprandial satiety, decreases in subsequent energy intake, and increased postprandial thermogenesis [9]. Increasing dietary protein specifically at breakfast rather than other mealtimes may be desirable because protein consumption is lower at breakfast compared to lunch and dinner, [10] and increases in dietary protein at breakfast have been previously shown to have greater effects on satiety compared to other mealtimes [11]. Limited data also suggest that higher protein breakfasts may decrease pre-lunch neural responses to visual food stimuli in reward-related brain regions including the insula and pre-frontal cortex [12]. Consumption of a higher protein breakfast may also confer reductions in neural responses to visual food stimuli throughout the day as evidenced by decreased pre-dinner hippocampal and parahippocampal responses following consumption of a high *vs.* normal protein breakfast [13].

Increasing dietary fiber consumption is another potentially promising strategy to prevent weight gain in overweight adults. Ninety-five percent of Americans are consuming less than the Adequate Intake for fiber, and fiber was identified as a nutrient of concern by the 2015 Dietary Guidelines Advisory Committee [14]. Increased fiber consumption is associated with decreased body weight, which may be partially explained by reductions in hunger and subsequent energy intake with higher fiber meals/diets [15]. However, the effects of fiber on subjective appetite, energy intake, and body weight are likely influenced significantly by the physiochemical properties of the fiber being studied [15], and at least one systematic review found that the majority of studies did not support a beneficial impact of fiber on appetite or energy intake, regardless of fiber-type [16].

Considering the relatively modest individual effects of dietary protein and fiber on body weight and ingestive behavior, interventions to increase both protein and fiber may be more effective for modifying appetite and ingestive behavior than either approach alone. However, the additive and/or interactive effects of dietary protein and fiber are understudied. Therefore, the purpose of this randomized, double-blind, crossover study was to evaluate the individual and combined effects of higher protein and fiber at breakfast on subjective appetite, neural responses to visual food stimuli, and *ad libitum* energy intake at lunch in overweight adults. We hypothesized that increasing either dietary protein or fiber at breakfast would increase postprandial fullness and decrease postprandial hunger, desire to eat, energy intake at lunch, and neural responses to visual foods stimuli, and that a high protein and high fiber breakfast would have an additive effect on these outcomes.

## 2. Experimental Section

### 2.1. Subjects

**Figure 1:**
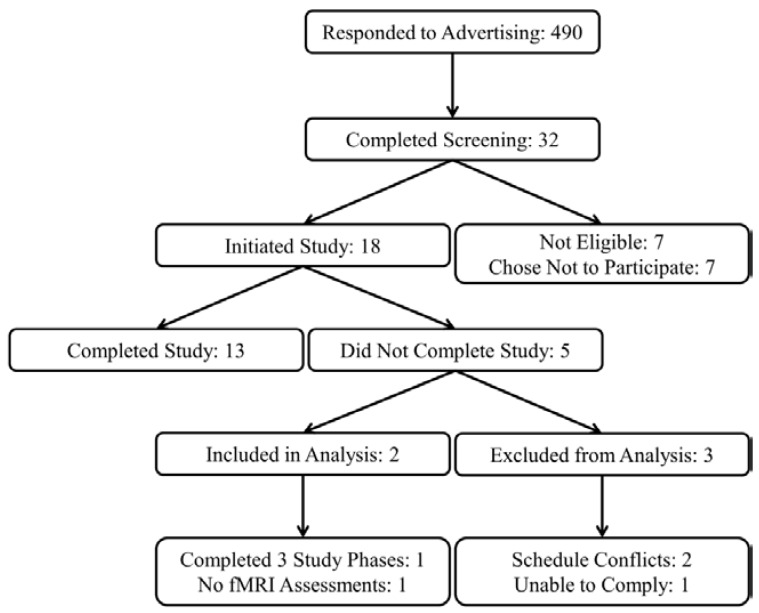
Study recruitment flow diagram.

Eighteen (6 female and 12 male) subjects were recruited from the greater Lafayette, IN community to participate in a research study at Purdue University, and 13 subjects completed all study procedures. Data from two subjects with incomplete study data were also included for statistical analysis resulting in a total sample of 15 subjects (5 female and 10 male, Figure 1).

Inclusion criteria for this study were: male or female; age 21–45 years; overweight BMI (25.0–29.9 kg/m^2^); regularly eating breakfast (≥5 days/week); weight stable (±3 kg for previous 6 months); not participating in organized sports or regular exercise (≥2 days/week); no tobacco use; no diabetes; not pregnant or lactating for 6 months; not claustrophobic; no implanted pacemakers/automated defibrillators or ferromagnetic metal. All subjects provided written informed consent and received a monetary stipend. The consent form and all study procedures and documents were approved for use by the Purdue University Biomedical Institutional Review Board. This trial was registered on ClinicalTrials.gov (NCT02169245).

### 2.2. Experimental Design

On four testing days after an overnight fast, subjects consumed one of the following breakfasts in random order: normal protein and normal fiber, normal protein and high fiber, high protein and normal fiber, and high protein and high fiber. Subjects and investigators were blinded to the protein and fiber content of the breakfasts until after statistical analyses were completed. Fasting-state subjective appetite assessments (hunger, desire to eat, and fullness) and functional magnetic resonance imaging (fMRI) were completed upon subject arrival at the Purdue University MRI Facility at 0700, 0800, or 0900 (−45 min). Additional appetite assessments were completed immediately before (−15 min) and after breakfast (0 min), 60, 120, 180, and 240 min after breakfast (also served as pre-lunch assessment), and immediately after consuming an *ad libitum* lunch. A postprandial fMRI scan was performed immediately after subjects completed the appetite assessment at the 180 min postprandial time point. Lunch was served immediately after subjects completed the 240 min appetite assessment and *ad libitum* energy intake at lunch was measured. Subjects were served an approximately 1000 kcal serving of pasta with marinara sauce in a room with limited distractions (no investigators, TV, radio, phone, *etc.*) and instructed to eat until they were comfortably full.

All testing days were separated by ≥26 days (mean: 30 days, range: 26–49 days) and subjects were provided with all breakfast meals for 2 weeks prior to each testing days (breakfast intervention period). The protein and fiber contents of the provided breakfasts matched that of the breakfast served on each testing day. Subjects were not given additional instructions or dietary advice and were allowed to choose and consume self-selected foods and beverages *ad libitum* for lunch, dinner, and snacks during each breakfast intervention period. A minimum 2-week washout period was completed after each testing day during which time subject consumed self-selected foods and beverages *ad libitum*.

### 2.3. Breakfast Characteristics

All breakfast meals were ~400 kcal and provided ~50 g of available carbohydrates. Normal and high protein breakfasts provided 12 g and 25 g of protein, respectively. The difference in protein content between the normal and high protein breakfasts was derived from whole eggs and/or egg white powder. The fat contents of the high protein breakfasts were decreased to maintain similar energy and carbohydrate amounts among all study breakfasts Normal and high fiber breakfasts provided 2 and 8 g of dietary fiber, respectively. The additional fiber in the high fiber breakfasts was achieved by the discrete addition of powdered psyllium husk to the meals. The resulting four breakfast interventions were normal protein + normal fiber (NPNF), normal protein + high fiber (NPHF), high protein + normal fiber (HPNF), and high protein +high fiber (HPHF). Four breakfast meal types (egg and potato casserole, quiche, breakfast sandwich, and breakfast burrito) were provided within each 2-week breakfast intervention period. The breakfast burrito was consumed for all testing days (Table 1). The meals were designed by the research dietitian to be indistinguishable in appearance and flavor between breakfast intervention periods.

**Table 1:** Breakfast Characteristics.

Nutrient	Normal Protein + Normal Fiber	Normal Protein + High Fiber	High Protein + Normal Fiber	High Protein + High Fiber
Energy (kcal)	396	387	397	386
Carbohydrate (g)	51	51	50	48
Sugar (g)	18	11	22	14
Total Fiber (g)	2	8	2	8
Soluble Fiber (g)	0	6	1	7
Insoluble Fiber (g)	2	2	1	1
Protein (g)	12	12	25	25
Total Fat (g)	16	14	10	10
Saturated Fat (g)	4	4	3	3
Monounsaturated Fat (g)	6	6	3	3
Polyunsaturated Fat (g)	3	2	1	1
Trans Fat (g)	0	0	0	0
Cholesterol (mg)	114	114	325	325
Sodium (mg)	767	765	723	720

### 2.4. Body Composition

Baseline body mass and % body fat were measured using the BOD POD Gold Standard Body Composition Tracking System (COSMED USA, Inc., Concord, CA, USA). Height was measured using a wall-mounted stadiometer (Holtain Ltd., Crymych, Wales, UK). Body mass index (kg/m^2^) was calculated at the beginning of the study using weight and height measurements.

### 2.5. Fasting-State Serum Glucose and Lipid Profile

Baseline fasting-state blood samples were collected from an antecubital vein into 3.5 mL serum-separator tubes, allowed to sit at room temperature for 30 min, and centrifuged for 15 min at 4400 rpm and 4 °C to obtain serum. Serum samples were sent to a professional laboratory (Mid America Clinical Laboratories, Lafayette, IN, USA) for analysis.

### 2.6. Appetite Assessments

Subjects rated their hunger, desire to eat, and fullness on continuous visual analog scales (VAS) [17] using Adaptive Visual Analog Scales software (Neurobehavioral Research Laboratory and Clinic, San Antonio, TX, USA) [18], which were completed on a laptop computer. Subjects were asked to report their level of hunger, desire to eat, and fullness by clicking on a continuous 100 mm scale with descriptors ranging from “Not at all” to “Extremely”.

### 2.7. fMRI Data Acquisition

Functional imaging was performed using a 3.0 Tesla magnetic resonance scanner (General Electric, Signa HDx, Milwaukee, WI, USA) while subjects were lying quietly in a supine position and presented with visual stimuli using NordicNeuroLab’s VisualSystem (Bergen, Norway). Visual stimuli consisted of images of food of high hedonic value and neutral nonfood-related objects, which were previously validated [19] and utilized in previously published reports [20,21,22,23,24,25]. Head movement was limited by placing foam pads behind the subjects’ necks and between the 16-channel head coil (Nova Medical, Inc., Model NMSC-025A, Wilmington, MA, USA) and all sides of the subjects’ heads. A localizer scan was prescribed and centered at the subjects’ brow line. The type, number and placement of foam pads, the location of subjects inside of the fMRI scanner, and the location of localizer prescription were noted during the first fMRI scanning session and replicated to the greatest extent possible for subsequent scanning sessions.

Three functional runs were performed during each fMRI session. Each run consisted of three blocks of visual food stimuli and three blocks of visual nonfood stimuli presented in a pseudorandomized order using PsychoPy, Version 1.76.00 [26]. Each block of visual stimuli lasted 30 s and included 10 images presented for 2.5 s each with a 0.5 s fade between each image. Blocks of a low-level baseline stimulus (fixation cross) lasting 16 s each were presented before the first visual stimuli block, in between blocks of visual stimuli, and after the final visual stimuli block.

Functional images were acquired with an echo-planar gradient-echo T2* blood oxygenation level dependent (BOLD) contrast sequence, with TR = 2000 ms, TE = 30 ms, 64^2^ matrix, 20 cm^2^ field of view, 40 slices to cover the whole brain, 3.1 mm think, and no gap between slices. A high resolution, T1-weighted anatomical scan was completed after functional imaging for coregistration with functional images.

### 2.8. fMRI Data Processing and Analysis

fMRI data preprocessing and first-level fMRI data analyses (food *vs.* nonfood BOLD contrasts) were completed using AFNI [27]. The first five volumes of each functional run (presented during the fixation cross block) were excluded to eliminate any T1 relaxation effects that may have been present due the relatively short TR of 2000 ms. Functional runs were then slice-time corrected, motion corrected to the first non-excluded image of the first functional run, smoothed using a 4.0 mm Gaussian blur, and the signal was normalized. Motion and slice-time corrected functional runs were then aligned with the high resolution anatomical scan.

Censor files were created to identify volumes within each functional run with excessive head motion (>2.5 mm). Those volumes that were censored were excluded and the six (roll, pitch, yaw, left-right, superior-inferior, anterior-posterior) motion time-series were included as covariates in the first-level, food *vs.* nonfood contrast regression model. The resulting food *vs.* nonfood contrast was expressed as a β coefficient for each fMRI session.

We first examined *a priori* regions of interest (bilateral insula, amygdala, orbitofrontal cortex, caudate, and putamen) to search for local maxima from the food *vs.* nonfood BOLD contrast during the fasting fMRI session on the first testing day. Spherical regions of interest with a 3 mm radii centered at the local maxima were then created to form each functional region of interest. Functional regions of interest obtained from the fasting-state fMRI scan on the first testing day were used for all subsequent fMRI scans.

### 2.9. Statistical Analyses

All statistical analyses were completed using SAS (Version 9.3, SAS Institute Inc., Cary, NC, USA) unless otherwise noted. Mean β coefficients (average of all voxels in each region of interest) representing the first-level, food *vs.* nonfood BOLD contrast from the fasting fMRI scan on the first testing day were analyzed using single-sample Student’s *t*-tests (PROC TTEST) to determine if the contrast was significantly different from zero. A Bonferroni correction was applied to correct for multiple comparisons among 10 *a priori* brain regions of interest (α = 0.05/10 = 0.005). Only regions of interest demonstrating a significant food *vs.* nonfood BOLD contrast in the fasting state on the first testing day were considered for further analyses.

Doubly repeated measures ANOVA (PROC MIXED) was used to assess the effects of protein (high *vs.* normal), fiber (high *vs.* normal), time (fMRI: fasting-state/−45 min *vs.* postprandial/180 min; appetite: pre-meal/−15 min *vs.* 0 min *vs.* 60 min *vs.* 120 min *vs.* 180 min *vs.* 240 min; repeated factor), and protein × time, fiber × time, and protein × fiber × time interactions on neural responses to visual food stimuli and ratings of hunger, desire to eat, and fullness. Chronological testing order (Day 1 *vs.* Day 2 *vs.* Day 3 *vs.* Day 4; repeated factor) and intervention carry-over effects were included in the model as covariates. Repeated measures ANOVA (PROC MIXED) was used to assess the effects of protein (high *vs.* normal), fiber (high *vs.* normal), protein × fiber interactions on *ad libitum* energy intake at lunch and total area under the curve (AUC) for postprandial hunger, desire to eat, and fullness. The statistical model included chronological testing order (Day 1 *vs.* Day 2 *vs.* Day 3 *vs.* Day 4; repeated factor) and intervention carry-over effects as covariates. AUCs for hunger, desire to eat, and fullness were calculated using the trapezoidal rule. A Tukey-Kramer adjustment for multiple comparisons was used as needed for *post-hoc* analyses. Linear correlations among appetite ratings, *ad libitum* energy intake, and neural responses to visual food stimuli were reported as Pearson’s correlation coefficients and calculated using a model that statistically accounted for repeated measurements among subjects (PROC MIXED). Data are presented as mean ± SEM and significance was set at α = 0.05 unless otherwise noted.

## 3. Results

### 3.1. Subject Characteristics

On average, subjects were 26 ± 1 years and overweight (BMI: 27.1 ± 0.2 kg/m^2^, 26.3 ± 2.4% body fat). Indicators of baseline metabolic health (fasting glucose and lipid profile) were within the normal reference ranges (Table 2).

**Table 2:** Baseline subject characteristics mean and standard error (SEM) (*n* = 15).

Parameter	Mean ± SEM
Age (year)	26 ± 1
Body Mass (kg)	82.3 ± 2.7
BMI (kg/m^2^)	27.1 ± 0.2
% Body Fat	26.3 ± 2.4
Glucose (mg/dL)	94 ± 2
Total Cholesterol (mg/dL)	164 ± 7
LDL-Cholesterol (mg/dL)	97 ± 7
HDL-Cholesterol (mg/dL)	48 ± 3
Triglycerides (mg/dL)	98 ± 10

Abbreviations: LDL, low density lipoprotein; HDL, high density lipoprotein.

### 3.2. Appetite Ratings and *ad Libitum* Energy Intake at Lunch

Fasting-state hunger (NPNF: 47 ± 1 mm, NPHF: 50 ± 1 mm, HPNF: 53 ± 1 mm, HPHF: 44 ± 2 mm; *p* = 0.15), desire to eat (NPNF: 51 ± 2 mm, NPHF: 51 ± 1 mm, HPNF: 56 ± 1 mm, HPHF: 48 ± 1 mm; *p* = 0.39), and fullness (NPNF: 24 ± 1 mm, NPHF: 22 ± 1 mm, HPNF: 22 ± 1 mm, HPHF: 22 ± 1 mm; *p* = 0.82) ratings were not influenced by the amount of protein and fiber consumed at breakfast during the preceding 2-week intervention.

**Figure 2:**
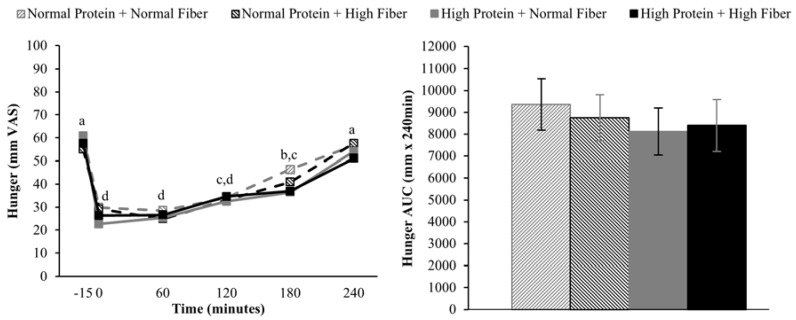
Postprandial time course (**left**) and area under the curve (AUC) (**right**) for hunger on the four testing days. Postprandial time points with different letters are statistically different (main effect of time, Tukey-adjusted *p* < 0.05).

**Figure 3:**
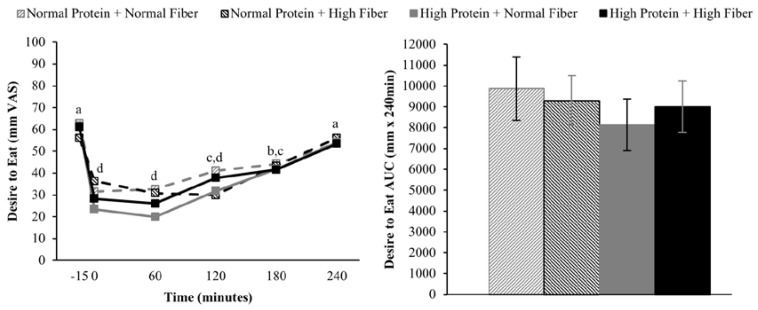
Postprandial time course (**left**) and AUC (**right**) for desire to eat on the four testing days. Postprandial time points with different letters are statistically different (main effect of time, Tukey-adjusted *p* < 0.05).

**Figure 4:**
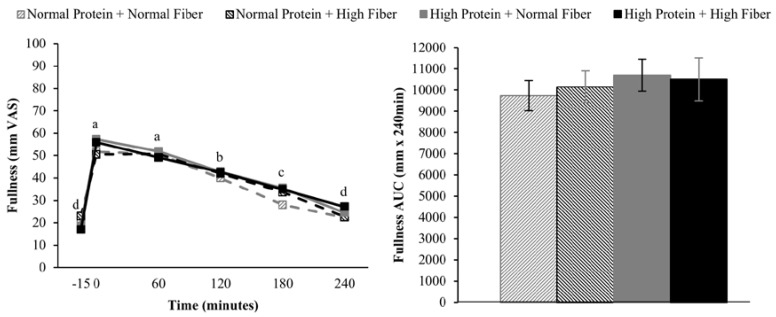
Postprandial time course (**left**) and AUC (**right**) for fullness on the four testing days. Postprandial time points with different letters are statistically different (main effect of time, Tukey-adjusted *p* < 0.05).

Compared to pre-meal (−15 min) ratings, hunger and desire to eat were decreased and fullness increased for 180 min following breakfast consumption (main effect of time), but appetite ratings 240 min after breakfast consumption were not different from fasting-state ratings (Figure 2, Figure 3 and Figure 4). The postprandial time courses and AUCs for hunger, desire to eat, or fullness were not influenced by protein or fiber amount (Figure 2, Figure 3 and Figure 4). *Ad libitum* energy intake at lunch was also not influenced by the protein and fiber content of the breakfasts (NPNF: 553 ± 46 kcal, NPHF: 548 ± 60 kcal, HPNF: 539 ± 57 kcal, HPHF: 520 ± 64 kcal; *p* = 0.94). Post-lunch hunger, desire to eat, and fullness ratings were not influenced by the breakfast type.

### 3.3. Neural Responses to Visual Food Stimuli

**Figure 5:**
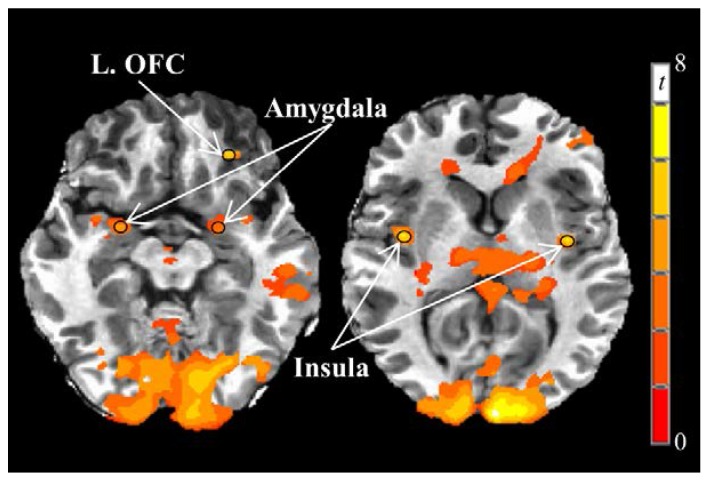
Fasting-state neural responses to visual food stimuli on Day 1. Greater responses to visual food stimuli *vs.* nonfood stimuli (PROC TTEST, SAS, Version 9.3; *p* < 0.005) were observed in the bilateral insula and amygdala and left orbitofrontal cortex. Black circles represent functional regions of interest with 3 mm radii within *a priori* brain regions of interest with known reward functions. Images are in the axial plane and left side of the figure corresponds to the right side of the body and *vice versa*. Display threshold: *p* < 0.001 (uncorrected), minimum cluster size of 250 voxels. Abbreviations: OFC, orbitofrontal cortex.

Greater fasting-state responses to food *vs.* nonfood visual stimuli on the 1st testing day were observed in the left and right insula, left and right amygdala, and left orbitofrontal cortex (Table 3, Figure 5), but not the left and right caudate, left and right putamen, or right orbitofrontal cortex. Compared to fasting-state, neural responses to visual food stimuli 180 min after breakfast were reduced (main effect of time) in the right insula (*p* = 0.044) and left amygdala (*p* = 0.01). Postprandial neural responses in the left insula (*p* = 0.065) and right amygdala (*p* = 0.076) also tended to be decreased relative to fasting-state.

**Table 3:** Responses to visual food cues compared to nonfood cues in the fasting-state on Day 1.

Brain Region	MNI Coordinates ^1^			*T* Value ^2^	*p* Value ^3^
	*x*	*Y*	*z*		
Insula (L)	−38	−7	7	6.22	<0.0001
Insula (R)	40	−5	4	5.94	<0.0001
Amygdala (L)	−23	0	−17	4.46	0.0006
Amygdala (R)	22	0	−20	4.20	0.0010
OFC (L)	−26	33	−18	4.12	0.0012

^1^ Stereotactic coordinates in MNI space for local maxima for the food *vs.* nonfood contrast within each brain region of interest; ^2^ *T* values reported represent the results of single sample *t*-tests for comparison of mean β coefficient of all voxels in each region for food *vs.* nonfood contrast to zero; ^3^ Uncorrected *p* values. Abbreviations: MNI, Montreal Neurological Institute; OFC, orbitofrontal cortex; L, left; R, right.

The breakfast intervention did not further influence postprandial neural responses in the amygdala or orbitofrontal cortex. Postprandial right insula responses were decreased relative to the fasting-state after consuming normal protein breakfasts (*p* = 0.030, Tukey adjusted) but not high protein breakfasts (*p* = 0.97, Tukey adjusted). *Post-hoc* analyses confirmed that postprandial right insula responses were lower after consuming normal *vs.* high protein breakfasts (*p* = 0.051, Tukey adjusted). A significant time x fiber interaction (*p* = 0.047) was observed for left insula responses such that responses were only reduced relative to fasting-state after consuming normal fiber breakfasts. However, post hoc analyses indicated that postprandial responses were not different following consumption of normal *vs.* high fiber breakfasts (*p* = 0.73, Tukey adjusted).

### 3.4. Correlations

*Ad libitum* energy intake at lunch was not linearly correlated with postprandial neural responses to visual food stimuli in any *a priori* brain regions of interest. Left and right insula and amygdala responses were not linearly correlated with any measure of subjective appetite. Left orbitofrontal cortex responses were positively, linearly correlated with hunger (*r* = 0.32, *p* = 0.003) and desire to eat (*r* = 0.25, *p* = 0.02) but not fullness (*r* = −0.16, *p* = 0.19).

## 4. Discussion

The primary aim of this study was to assess whether altering the amount of protein and/or fiber consumed at breakfast would influence acute ingestive behavior as measured via subjective appetite ratings, neural responses to visual food stimuli, and *ad libitum* energy intake at lunch. Contrary to our hypotheses, the discreet manipulation of protein and fiber at breakfast had only limited effects on acute ingestive behavior outcomes. More traditional methods of investigating ingestive behavior (subjective appetite ratings and *ad libitum* energy intake) were not influenced by protein or fiber. Contrary to expectations, results from this study suggest that postprandial right insula responses were suppressed by normal protein, but not the high protein breakfasts.

The utilization of fMRI and visual food stimuli to investigate neural reward influences on ingestive behavior has increased substantially in recent years, especially since 2009 [28]. The selection of *a priori* reward-related brain regions of interest for the current study was informed by meta-analytical findings of activation to food *vs.* nonfood visual stimuli in the insula, striatum, amygdala, and orbitofrontal cortex of normal weight adults [29]. Further, overweight/obese individuals have been consistently shown to demonstrate greater responses to visual food stimuli in the same brain regions compared to normal weight individuals [28,30]. In the current study and using a block design, visual food stimuli presented in the fasting state on the first testing day elicited greater responses compared to nonfood stimuli in 5 of 10 *a priori* regions of interest (left and right insula, left and right amygdala, and left orbitofrontal cortex).

Collectively, these brain regions work interactively to encode reward value and influence motivated behavior [30]. More specifically, the insula interprets current, previous, and predicted bodily states through interoception that guide behavioral decision making. The insula is also characterized by a high density of dopamine and µ-opioid receptors that contribute to its role in reward processing [31,32]. The amygdala has long been associated with encoding fear and encouraging avoidance behavior. However, the amygdala is now known to be heavily involved in positive affect as well and interacts with the orbitofrontal cortex and sensory regions to adjust motivational state [33,34]. The orbitofrontal cortex receives inputs from numerous brain regions including the anterior cingulate cortex, amygdala, and hippocampus, which support its role in integrating reward-related cues and making informed outcome predictions [35,36]. While the insula and amygdala responses were not associated with subjective measures of appetite in the current study, observed linear correlations between the left orbitofrontal cortex and hunger/desire to eat support its roles in encoding the reward value of visual food stimuli.

Previous research has demonstrated that neural responses to visual food cues are attenuated following meal consumption in normal weight adults. However, neural responses in the insula, amygdala, orbitofrontal cortex, and other reward-related brain regions have been reported to be relatively unaffected by meal consumption in obese adults [28]. Our results in overweight young adults are probably most comparable to those of Leidy *et al*. [12], which reported meal-induced reductions in responses to visual food stimuli in the hippocampus, amygdala, cingulate, and parahippocampus 3 h after eating breakfast compared to breakfast skipping. Furthermore, a high protein breakfast reduced insula and middle prefrontal cortex responses compared to a normal protein breakfast [12]. In the current study, however, postprandial right insula responses were lower after consuming normal protein compared to high protein breakfasts.

While the current and Leidy *et al.* [12] studies both included individuals in the overweight category and completed postprandial fMRI scanning 3 h after consuming breakfast, a number differences in study designs exist that may have contributed to the conflicting findings. Subjects in the current study were males and females aged 26 years on average, while Leidy *et al.* recruited only females with an average age of 15 years [12]. Another fundamental difference between the two studies is that Leidy *et al.* sought to study individuals who regularly skip breakfast (~5 times/week) [12], while the current study used regular breakfast consumption (defined as ≥5 times/week) as an inclusion criteria for study participation. Other notable differences between the two studies include the energy content of the meals (400 *vs.* 490 kcals), protein amounts (NP: 12 g *vs.* 18 g; HP: 25 g *vs.* 50 g), lack of “breakfast skipping” condition in the current study, and employment of a double-blind design with respect to protein (and fiber) amounts in the current study [12]. Given the complexity of energy balance regulation [37] and paucity of extant data on the influence of meal composition on postprandial neural reward processing, further research is necessary to bring clarity to the role of dietary protein and other nutrients in the modulation of reward-driven eating behaviors.

Increasing the protein content (and concomitantly decreasing the fat content) of the breakfast meals did not influence subjective appetitive sensations or *ad libitum* energy intake at lunch served 4 h after breakfast completion. The effects of higher protein consumption on postprandial appetite sensations are mixed, but in general, higher protein meals do appear to have a modest beneficial effect on satiety [9,38]. However, it is important to note that the majority of studies demonstrating increased satiety/fullness and/or decreased hunger and desire to eat have utilized protein amounts that were greater than the current study (≥40% *vs.* 25% protein as a percentage of total energy) [9]. Although not rigorously tested, a protein quantity threshold of 30 g has been proposed to be necessary to elicit a satiety effect [38]. If this is the case, the 25 g high protein meal served in the current study may not have been sufficient to alter postprandial appetite sensations.

Similar to protein, the addition of ~6 g of fiber as psyllium husk powder at breakfast did not influence subjective appetite sensations or *ad libitum* energy intake at lunch. Despite a widespread belief that fiber enhances satiety, empirical data supporting this claim are inconsistent and mixed [15,16]. One systematic review concluded that higher fiber diets/meals do not consistently modulate appetite sensations and ingestive behavior, regardless of fiber type [16]. Another systematic review, on the other hand, concluded that more viscous fibers were more effective than less viscous fibers at reducing appetite and energy intake [15]. The additional 6 g of fiber in the high fiber breakfast meals in the current study was derived from psyllium husk powder, which is classified as a viscous fiber [15]. It is possible that difference in fiber between the normal and high fiber breakfasts (2 g *vs.* 8 g) may not have been sufficient to elicit differential appetite and ingestive behavior responses. However, data regarding the effect of fiber dose on perceived satiety and *ad libitum* energy intake are unclear. For example, fiber doses ranging from 5.0–7.4 g to 10.0–14.9 g appear to be most effective for increasing perceptions of satiety, but doses of ≥30 g may be necessary to affect energy intake [16].

Strengths of the current study include the use of double-blind study design that would be expected to limit potential biases of both the subjects and investigators, which is especially important for a study with subjective variables such as appetite ratings. Physiologically relevant *a priori* brain regions of interest with a history of responsiveness to visual food stimuli paradigms were chosen for the current study. The addition of objectively measured *ad libitum* energy intake at lunch represents another strength of the current study that is commonly lacking from other fMRI studies.

Primary limitations of the current study include a relatively small sample size comprised of a homogenous study population (young adults, overweight BMI). Therefore, caution is warranted when interpreting and generalizing the results of this study more widely to the general population. Some additional limitations of the study design should be considered when interpreting the results of the current study. In particular, increases in the protein content of the breakfasts were achieved via reductions in fat contents to maintain equivalence in total energy and carbohydrate content. Given the potential influence of dietary fats on appetite [39], it is not possible to definitively attribute the lack of changes in acute ingestive behavior to increases in protein, decreases in fat, or some combination of the two. A number of gut-derived peptides are known in influence ingestive behavior [40], which were not measured in the current study. Future studies should include measurements of these peptides to better delineate the physiological mechanisms controlling diet-induced modulation of ingestive behavior. In the current study, subjects consumed the respective breakfast type (NPNF, NPHF, HPNF, HPHF) daily for 2 weeks prior to the acute feeding trial, which may have resulted in some adaptation to the protein and/or fiber content of the breakfasts. However, higher protein breakfasts were previously shown to decrease hunger and desire to eat compared to normal protein breakfasts even after chronic consumption of a high-protein diet [41]. Lastly, we did not control food intake at lunch, dinner, and/or snacks on the day prior to the acute feeding trial, which could influence perceptions of appetite and ingestive behavior. We did not observe any differences in fasting-state hunger, desire to eat, or fullness ratings, which provides some confidence that the subjects’ self-selected food intake prior to testing days did not influence our results.

## 5. Conclusions

In conclusion, discreetly and modestly increasing protein and/or fiber amounts at breakfast may not be effective strategies for modulating acute ingestive behavior in overweight young adults. More profound alterations to protein and fiber intakes may be necessary to achieve changes in acute ingestive behavior, but the practicality of adopting and sustaining such large changes in eating behavior must be considered.
